# Effectiveness of hyaluronic acid and its derivatives on diabetic foot ulcer: a systematic review and meta-analysis

**DOI:** 10.3389/fendo.2025.1627558

**Published:** 2025-09-26

**Authors:** Ling Yao, Qinci Xie, Jiezhi Dai, Gaofeng Huang

**Affiliations:** ^1^ Department of Orthopedic Surgery, The Affiliated Hospital (GROUP) of Putian University, Putian, Fujian, China; ^2^ Department of Orthopedic Surgery, Jinjiang Municipal Hospital, Shanghai Sixth People’s Hospital Fujian, Jinjiang, Fujian, China; ^3^ Department of Orthopedic Surgery, Shanghai Sixth People’s Hospital, JiaoTong University, Shanghai, China

**Keywords:** diabetic foot, hyaluronic acid, wound healing, meta-analysis, chronic wound

## Abstract

**Background:**

In this study, we aim to evaluate the effects of hyaluronic acid and its derivatives on wound healing in diabetic foot ulcer.

**Methods:**

The electronic databases included PubMed, BIOSIS, EMBASE, Cochrane Central Register of Controlled Trials (CENTRAL), and Google Scholar internet. The final search was updated on Aug 31, 2024. We assessed eligible studies that comparing the effects of hyaluronic acid and its derivatives with other dressings on wound healing in diabetic foot ulcer. The primary outcomes included the rate of ulcers completely healed, time to healing and adverse event. The standard mean differences (SMDs) or the odds ratios (ORs) were calculated for continuous or dichotomous data, respectively. Data were analyzed by using the Cochrane Collaboration’s RevMan 5.0 software.

**Results:**

We assessed each included study with the Cochrane ‘Risk of bias’ tool. Seven RCTs involving 444 patients and 456 ulcers were included in our study. Hyaluronic acid may improve the complete ulcer healing rate (OR 3.92, 95% CI 1.74 to 8.81, P = 0.02, I^2^ = 62%) and shorten the time to ulcer healing (SMD = -0.83, 95% CI -1.13 to -0.53, P = 0.24, I^2^ = 28%), with no increasing the incidence of adverse events (OR = 0.79, 95% CI 0.46 to 1.35, P = 0.31, I^2^ = 16%).

**Conclusion:**

In conclusion, HA and its derivatives could be a potentially beneficial therapy for DFU treatment that promotes the complete ulcer healing rate, shortens healing time, without increasing incidence of adverse events.

**Systematic review registration:**

https://www.crd.york.ac.uk/prospero/display_record.php?ID=CRD42024588743, identifier CRD42024588743.

## Introduction

Diabetic foot ulcer (DFU) is the most serious and costly complication of diabetes (1). It plays a very important role in the occurrence of vascular disease, neuropathy, and infection of diabetes. In severe cases, amputation is required, which significantly affects the patients’ quality of life.

Wound dressing plays an important role in the clinical treatment of DFU. A kind of dressing with good hemostatic maintenance ability, anti-infection and promoting repair ability may be suitable for diabetic wound (2). In recent years, several studies have reported satisfactory results after the treatment of chronic complex wounds, with advanced dressings, including gauze, films, foams or, hydrocolloid-based dressings as well as polysaccharide- and polymer-based dressings (3).

Hyaluronic acid (HA) is a major component of the extracellular matrix that possesses desirable properties such as biocompatibility, biodegradability, and hydrophilicity (4). It has been found to be a promising candidate to promote wound healing by stimulating the proliferation of fibroblast, keratinocyte migration, and remodeling of the extracellular matrix (5). A recent Cochrane review by Roehrs et al. (6) evaluated the effects of HA and its derivatives on the healing of chronic wound, and found that HA probably improves complete ulcer healing and may increase change in ulcer size when compared with neutral vehicle. In particular, the effect of HA on diabetic wound remains unclear. Therefore, we presented a systematic review and meta-analysis to evaluate the effectiveness of HA and its derivatives on wound healing in DFU.

## Method

The systematic review and meta-analysis was conducted according to the Preferred Reporting Items for Systematic Review and Meta-Analyses (PRISMA) 2020 guidelines (7). It was registered at PROSPERO (https://www.crd.york.ac.uk/prospero/display_record.php?ID=CRD42024588743).

The electronic databases included PubMed, BIOSIS, EMBASE, Cochrane Central Register of Controlled Trials (CENTRAL), and Google Scholar internet. The final search was updated on Aug 31, 2024. No restriction of language was performed in this study. The following combination of search terms was used: (hyaluronic acid, or hyaluronate) AND (diabetic foot, diabetic wound, or DFU). We reviewed references from the original trials, grey literatures, and review articles to identify potential eligible articles. Two reviewers conducted literature searches independently and resolved differences through discussion with the third author.

### Inclusion and exclusion criteria

The inclusion criteria included: (1)randomized controlled trials (RCTs) that comparing the effects of hyaluronic acid with no hyaluronic acid on the healing of DFU; (2)skeletally mature patients, aged 18 or older with DFU; (3)patients treated with any type of wound dressing containing hyaluronic acid or any of its derivatives (zinc hyaluronate, HA hydrogels, or HA sponge, etc.) defined as the treatment group (HA group), and participants in the control treatment arm who had any other type of dressing, topical agent, placebo, or standard treatment (control group); (4)outcomes including complete ulcer healing rate at 12 weeks, time to healing, and adverse event (e.g. the presence of wound infection, inflammation, and worsening of ischemia).

The exclusion criteria included: (1) animal experiments and case reports; (2)the data was incomplete.

### Data extraction

Two reviewers independently screened the literatures, extracted data from the included trials, and consulted a third author when the two reviewers had disagreements. The extracted data included authors name, study design, publication year, country, sample size, age, sex, intervention program, follow-up time, and outcomes.

### Statistical analysis

Study analyses were performed with the Cochrane Collaboration’s RevMan 5.0 software. For dichotomous data (rate of ulcer healing and adverse event), we used odds ratio (OR) with 95% confidence intervals (CIs) to measure outcomes. For continuous data (time to healing), the standardized mean difference (SMD) with 95% confidence intervals (CIs) was used to measure outcomes. Heterogeneity among studies was evaluated using the chi-square tests (with P less than 0.05 representing heterogeneity) and the I^2^ statistic (with I^2^ more than 50% indicating high heterogeneity). A random effects model analysis was used when significant heterogeneity was found.

A sensitivity analysis was conducted by repeating the analysis after sequential exclusion of one study at a time. Publication bias was evaluated by visual inspection of funnel plots, the Begg’s rank correlation test (8), and the Egger’s regression test (9). Statistical analyses were tested using STATA 14.1 software. A P value less than 0.05 was considered statistically significant.

Two authors independently assessed the risk of bias with the Cochrane Risk of Bias Tool for Randomized Controlled Trials (RCTs) (10). The assessment tool addressed five main fields included sequence generation, allocation concealment, blinding, incomplete outcome data and selective outcome reporting.

## Result

**Figure 1:**
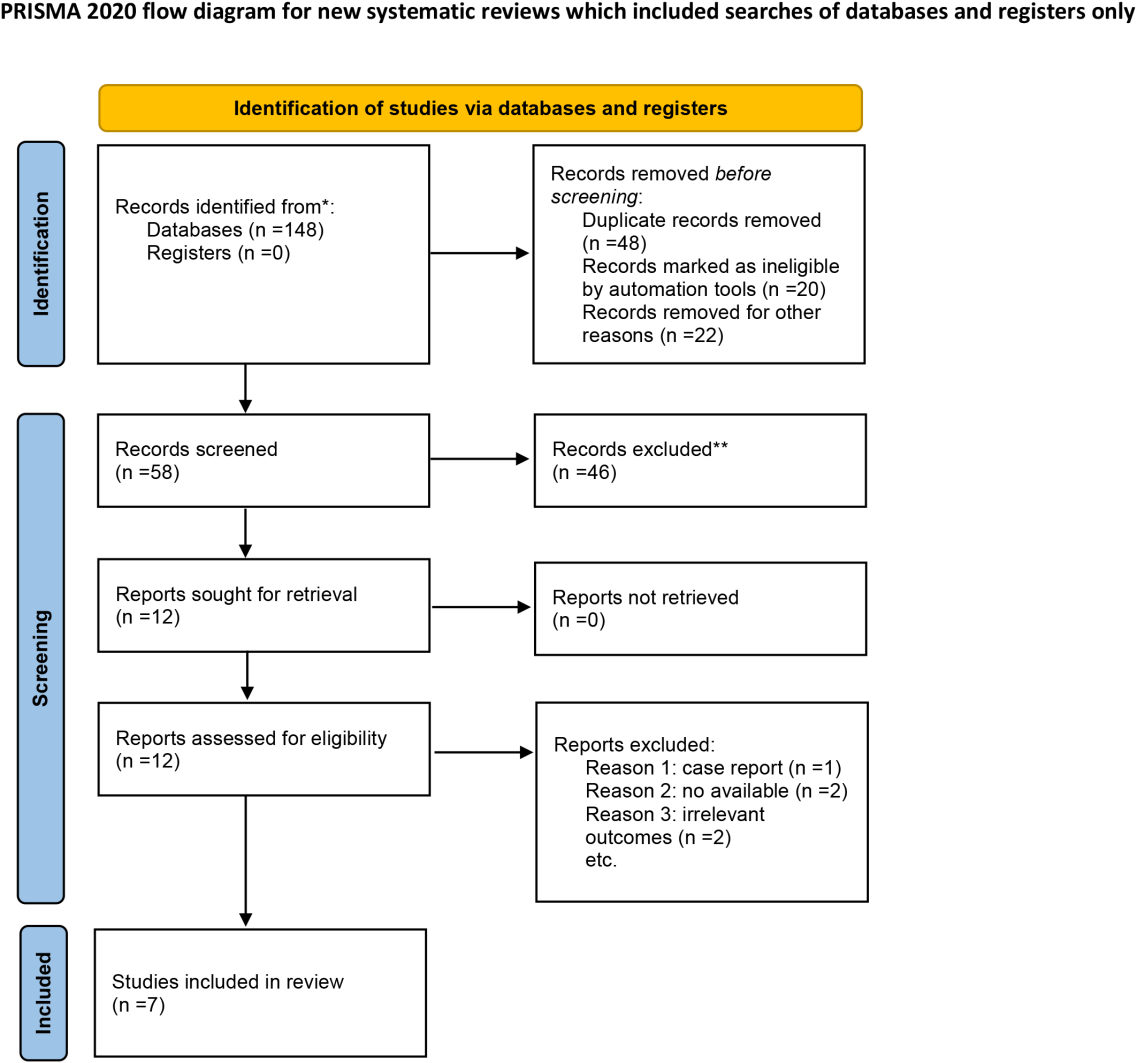
Flow diagram for study selection. *Consider, if feasible to do so, reporting the number of recards identified from each database or register searched (rather than the total number across all databases/registers). **If automation tools were used, indicate how many records were excluded by a human and how many were excluded by automation tools.

**Figure 2:**
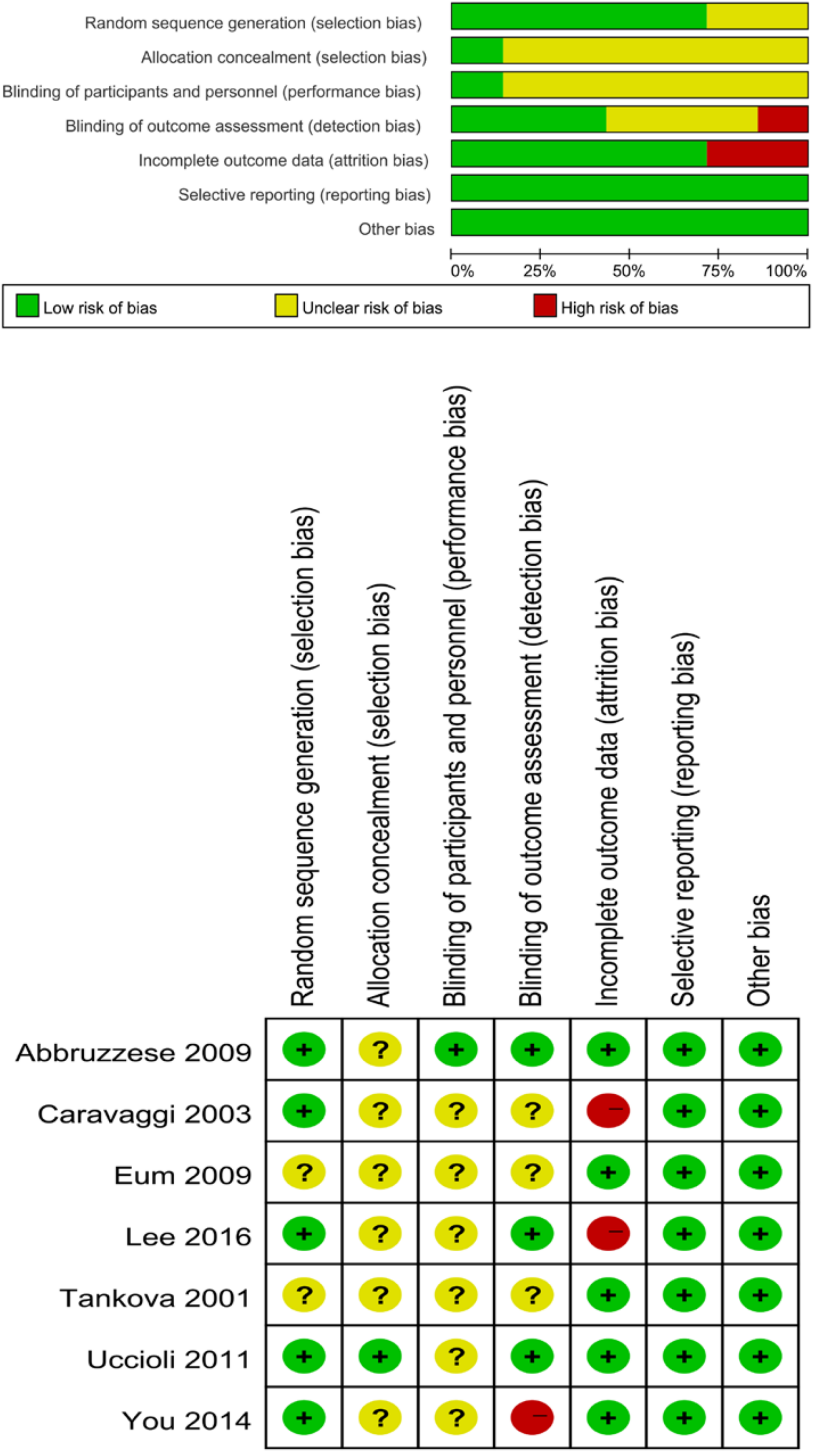
Quality evaluation of the included studies.

A total of 82 primary studies were identified from online databases prior to Aug 2024. The process of study selection is reported in **Figure 1**. According to the inclusion and exclusion criteria, seven RCTs involving 444 patients and 456 ulcers were included in our study (11–17). All were published in English. The sample ranged from 25 to 160. Three articles were published in Italy, three were from Korea, and one was from Bulgaria. Detailed of included RCTs are presented in **Table 1**. The outcomes of included studies are reported in **Table 2**. The qualities of included studies are shown in **Figure 2**.

**Table 1:** The characteristics of the included studies.

Study	Country	Sample size		Mean age (years)		Sex(m/f)		Treatment strategy		Size of ulcer (cm2)		Ulcer duration (weeks)		Follow-ups
		H	C	H	C	H	C	H	C	H	C	H	C	
Abbruzzese 2009(13)	Italy	15	15	61.8	62.4	NR	NR	HA gel	Conventional treatment	25.9 ± 8.8	27.3 ± 10.4	30.8 ± 16.7	22.9 ± 18.6	12w
Caravaggi 2003 (12)	Italy	43	36	NR	NR	NR	NR	Hyalograft3D+ autograft	Conventional treatment	5.3 ± 6.76	6.2 ± 7.58	16 ± 40	16 ± 24	12w
Eum 2009 (14)	Korea	14	14	NR	NR	NR	NR	Hyalograft3D+ autograft	Conventional treatment	3.9 ± 4.38	3.9 ± 2.02	46 ± 94.9	26 ± 20.64	NR
Lee 2016 (17)	Korea	13	12	57.08	57.58	11/2	8/4	HA dressing	Conventional treatment	3.1 ± 2.48	4.8 ± 4.32	18.53 ± 5.82	17.66 ± 4.51	24w
Tankova 2001 (11)	Bulgaria	35a	24b	55.7		37/22		Zinc hyaluronate	Conventional treatment	10.32 ± 4.6	11.46 ± 5.4	26.8 ± 16.8		12w
Uccioli 2011 (15)	Italy	80	80	61	62	NR	NR	Hyalograft3D+ autograft	Conventional treatment	8.8 ± 9.4	6.7 ± 7.7	7.4 ± 6.6	7.3 ± 7.8	20w
You 2014 (16)	Korea	31	32	61.2	63.8	21/10	22/10	Hyaluronic acid sheet+ autograft	Conventional treatment	3.5 ± 3.7	2.9 ± 2.7	24.4 ± 65.6	24.8 ± 78.8	12w

H, HA group; C, Control group; a: 43ulcers; b: 28ulcers; Hyalograft3D, hyaluronic acid based scaffold; NR, Not report

**Table 2:** The outcomes of the included studies.

Study	Time to healing (days)		Number of ulcer healing		Adverse event	
	H	C	H	C	H	C
Abbruzzese, 2009(13)	60.4 ± 24.8	79.9 ± 18.6	14	9	4	5
Caravaggi, 2003	NR	NR	28	18	7	10
Eum, 2009 (14)	42.56 ± 24.49	78.44 ± 17.02	12	4	NR	NR
Lee, 2016 (17)	NR	NR	11	5	1	4
Tankova, 2001 (11)	74 ± 31	92 ± 25	32	20	1	2
Uccioli, 2011(15)	NR	NR	40	34	18	14
You, 2014 (16)	36.4 ± 17.6	48.4 ± 13.1	26	11	NR	NR

H, HA group; C, Control group; NR, Not report

**Figure 3:**
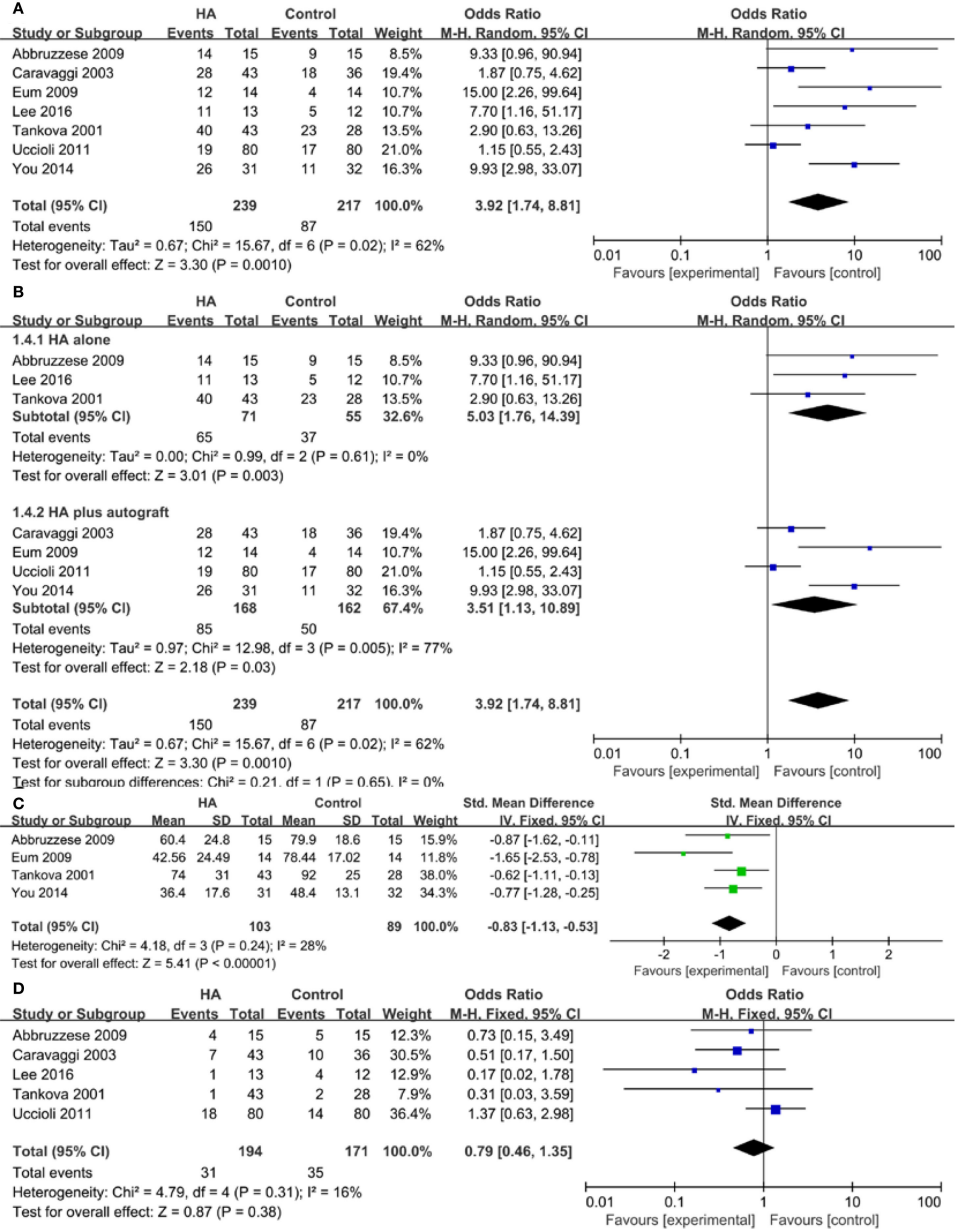
Meta-analysis of complete ulcer healing rate **(A)**, subgroup analysis of complete ulcer healing rate **(B)**, time to healing **(C)**, and adverse events **(D)**.

**Figure 4:**
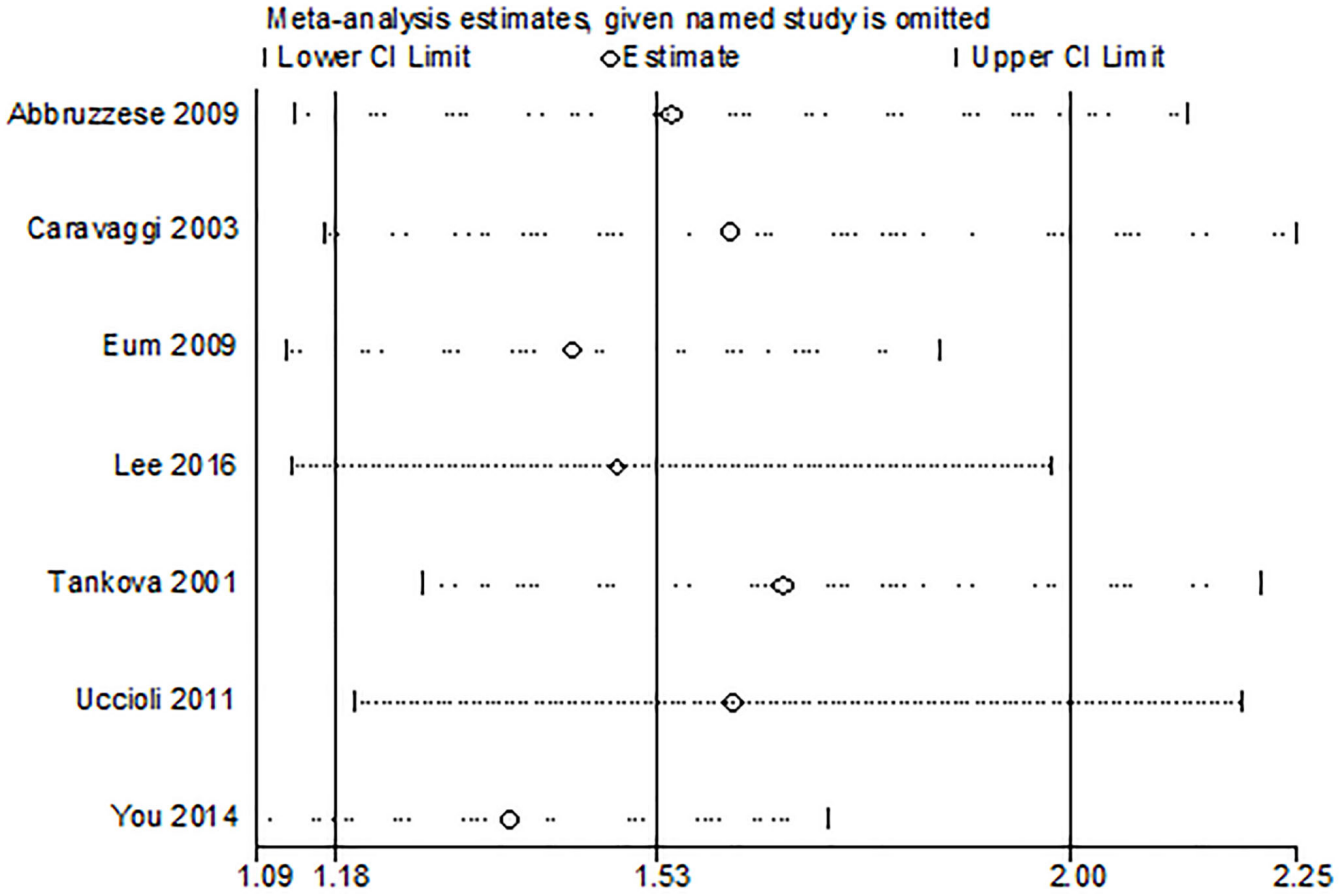
Sensitivity analysis of the meta-analysis.

Seven trials reported the outcome of ulcer healing rate. The study showed a higher ulcer healing rate in the HA group compared with the control group (OR 3.92, 95% CI 1.74 to 8.81, I^2^ = 62%). There was a significant heterogeneity among these studies (I^2^ = 62%, P = 0.02) (**Figure 3A**). Results gave a pooled rate of 62.76% (150/239) in the HA group and of 40.09% (87/217) in the control. We performed a sensitivity analysis and the result showed that summary results were not significantly influenced by any single study (**Figure 4**). In addition, we performed a subgroup analysis based on the HA intervention (HA alone and HA plus autograft). Subgroup analysis indicated that the rate of complete ulcer healing varied with the HA intervention (HA alone OR = 5.03, 95% CI 1.76 to 14.39, P = 0.61; HA plus autograft OR = 3.51, 95% CI 1.13 to 10.89, P = 0.005) (**Figure 3B**). Regarding HA intervention, I^2^ changed from 0% for HA alone group to 77% for HA plus autograft group. Different HA intervention might not account for the heterogeneity.

Four studies reported the data of time to healing. A lower wound healing duration was found in the HA group compared with the control group (SMD = -0.83, 95% CI -1.13 to -0.53, I^2^ = 28%). There was no between-study heterogeneity among these studies (P = 0.24, I^2^ = 28%) (**Figure 3C**).

Five trials reported the outcome of adverse events. 31/194 participants (15.98%) in the HA group experienced an adverse event compared with 35/171 (20.47%) in the control group. The overall pooled data showed no significant difference (OR = 0.79, 95% CI 0.46 to 1.35, P = 0.31, I^2^ = 16%) (**Figure 3D**).

**Figure 5:**
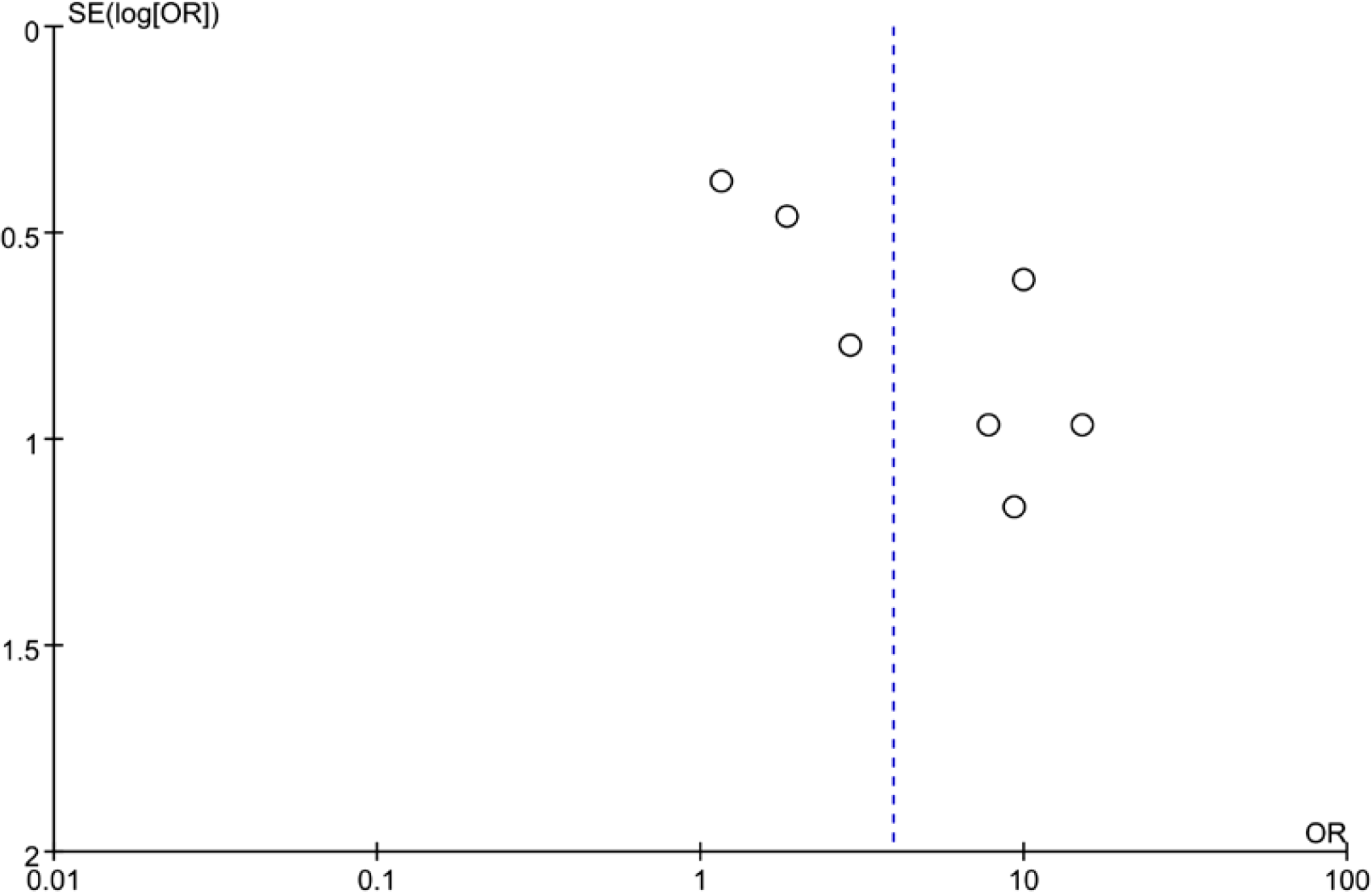
Publication bias analysis of the meta-analysis.

The funnel plot in the meta-analysis showed no evidence of publication bias in relation to risk of ulcer healing rate (**Figure 5**). It was also proved by Egger’s test (P = 0.222) and Begg’s test (P = 0.368).

## Discussion

This meta-analysis evaluated the effect of HA and its derivatives on wound healing in DFU. When compared with standard treatment, HA and its derivatives significantly improved the complete ulcer healing rate, shorten healing time, without increasing incidence of adverse events.

Hyaluronic acid is a straight chain, glycosaminoglycan polymer of the extracellular matrix composed of repeating units of the disaccharide (18). Previous studies have reported its significant role in promoting wound healing and regulating immune inflammatory response (19, 20). A meta-analysis on the effect of HA on the healing of burns, epithelial surgical wounds, and chronic wounds reported improved healing when compared with traditional therapies or placebo (21). In the early stage of wound healing, cells secrete a large amount of HA, which can promote wound contraction, increase the activity of neutrophils, and accelerate the phagocytosis of necrotic tissue and bacteria (22). HA can also induce cell aggregation and promote the formation of blood vessels within the collagen and fibrin matrix, thus promoting wound healing (23). With its inherent biocompatibility, viscoelastic properties, biodegradability, and non-immunogenicity, HA make it an excellent candidate for diabetic wound dressing components (24).

In 2014, a meta-analysis by Chen et al. (25) reported the effectiveness of HA for treating diabetic foot. The authors suggested that HA was beneficial in treating diabetic foot by increasing the rate of wound healing. This meta-analysis only included four trials, and studies did not reported the outcome of time to complete healing. In this present study, we included three extra trials and performed an updated meta-analysis of RCTs. In addition to the result of ulcer healing rate, we compared the clinical outcomes on time to healing and incidence of adverse events. More comparison of outcomes can help us make better clinical decisions.

Different HA-based wound dressing are used in our study. It can either be placed directly on the wound or used as a substrate for future autologous tissue grafts. Three trials used hyaluronic acid based scaffold (Hyalograft3D) plus autograft. One study used hyaluronic acid sheet plus autograft. The other three studies used HA gel, HA dressing, and Zinc hyaluronate, respectively. We further performed a subgroup analysis. Both the two subgroup results revealed a significant difference in favor of HA for the healing rate (OR = 5.03, 95% CI 1.76 to 14.39, P = 0.61; OR = 3.51, 95% CI 1.13 to 10.89, P = 0.005). HA alone or in combination with other compounds has a positive wound healing effect in DFU. In recent years, different methods of HA for the production of different types of wound dressing, including hydrogels, films, scaffolds, foams, and topical formulations, and nanoformulations, have been widely used for wound management (26). An extensive investigation will be needed to develop and optimize these novel wound dressings.

Some limitation should be noted. First, we only included seven studies, and the sample size in most of the studies were small. Secondly, some trials did not report the clinical characteristics of the individuals included and ulcer type and stage, which might cause potential bias in the selection of participants. Thirdly, this review found moderate inter-study heterogeneity (I^2^ = 62%). We performed a sensitivity analysis by investigating the effect of each individual study on the pooled effect size, and the summary results were comparatively reliable. Each trial varied in terms of demographic factors, patient profile, ulcer size, location method of adding HA to the dressing material, and duration of use, which might contribute to the heterogeneity. In future, more RCTs are needed to support these findings.

In conclusion, HA and its derivatives could be a potentially beneficial therapy for DFU treatment that promotes the complete ulcer healing rate, shortens healing time, without increasing incidence of adverse events. The use of different HA-based wound dressing methods should be further assessed. In addition, high quality of RCTs are needed in the future.

## Data Availability

The raw data supporting the conclusions of this article will be made available by the authors, without undue reservation.
